# Apparent diffusion coefficient values of the white matter in magnetic resonance imaging of the neonatal brain may help predict outcome in congenital cytomegalovirus infection

**DOI:** 10.1007/s00247-023-05838-9

**Published:** 2024-01-06

**Authors:** Caroline Vande Walle, Annelies Keymeulen, Anna Oostra, Eva Schiettecatte, Ingeborg Dhooge, Koenraad Smets, Nele Herregods

**Affiliations:** 1 Department of Radiology and Nuclear Medicine, Ghent University Hospital, C. Heymanslaan 10, 9000 Ghent, Belgium; 2 Department of Neonatology, Ghent University Hospital, Ghent, Belgium; 3 Center for Developmental Disorders, Ghent, Belgium; 4 Department of Otorhinolaryngology, Ghent University Hospital, Ghent, Belgium

**Keywords:** Brain, Cytomegalovirus infection, Hearing loss, Follow-up studies, Magnetic resonance imaging, Newborn, White matter

## Abstract

**Background:**

White matter change is a well-known abnormality in congenital cytomegalovirus (cCMV) infection, but grading remains challenging and clinical relevance unclear.

**Objective:**

To investigate if quantitative measurement of white matter apparent diffusion coefficient (ADC) values in magnetic resonance imaging (MRI) of the neonatal brain can predict outcome in cCMV.

**Materials and methods:**

A retrospective, single-center observational study, including patients with cCMV who had a neonatal brain MRI with diffusion-weighted imaging, was performed between 2007 and 2020. Regions of interest were systematically placed in the white matter on the ADC maps. Two pediatric radiologists independently scored additional brain abnormalities. Outcome measures were neonatal hearing and cognitive and motor development. Statistical analysis included simple and penalized elastic net regression.

**Results:**

Neonatal brain MRI was evaluated in 255 patients (median age 21 days, 25–75 percentiles: 14–28 days, 121 male). Gyral abnormalities were noted in nine patients (3.5%), ventriculomegaly in 24 (9.4%), and subependymal cysts in 58 (22.7%). General white matter ADC was significantly higher in patients with neonatal hearing loss and cognitive and motor impairment (*P*< 0.05). For neonatal hearing loss, simple logistic regression using only general white matter was the best prediction model, with a receiver operating characteristic area under the curve (AUC)=0.76. For cognitive impairment, interacting elastic net regression, including other brain abnormalities and frontoparietal white matter ADC, performed best, with AUC=0.89. For motor impairment, interacting elastic net regression, including other brain abnormalities and deep anterior frontal white matter performed best, with AUC=0.73.

**Conclusion:**

Neonatal white matter ADC was significantly higher in patients with clinical impairments. Quantitative ADC measurement may be a useful tool for predicting clinical outcome in cCMV.

**Graphical Abstract:**

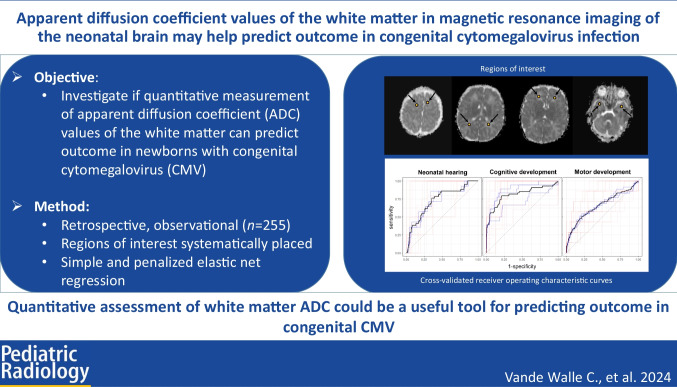

**Supplementary Information:**

Supplementary material is available at 10.1007/s00247-023-05838-9.

## Introduction

Congenital cytomegalovirus (cCMV) infection, being the leading type of congenital infection, causes a major health problem. Infected newborns have a significant risk of developing long-term impairments, mainly sensorineural hearing loss and neurodevelopmental problems [1–3].

At birth, only 10% of children show clinical signs of infection. Diagnosis in asymptomatic infants is often the result of testing for maternal seroconversion during pregnancy. Clinically symptomatic newborns have a very high risk of developing long-term sequelae (40–60%), although important developmental impairments are also seen in 10–15% of initially asymptomatic children [4–10].

Cytomegalovirus is a neurotropic virus. Infection in utero can cause a wide spectrum of brain anomalies, the type of lesion depending on the gestational age at the time of infection [11–14]. White matter abnormalities are one of the most characteristic findings of cCMV, occurring in up to 75% of clinically symptomatic and 30% of asymptomatic newborns. These lesions are often seen in association with other brain abnormalities; however, they are also frequently noted as an isolated finding [15, 16].

Despite the long-term impact of cCMV, evidence on which to base treatment decisions remains limited. According to the European consensus, patients with neuroimaging consistent with cCMV are considered “severely” symptomatic, and 6 months of antiviral therapy is recommended [17]. Magnetic resonance imaging (MRI) in newborns has shown a lot of promise complementary to ultrasound in detecting cCMV-related lesions, especially white matter abnormalities. Nevertheless, presently there is no consensus for its role in screening infected newborns, especially in the absence of clinical symptoms [14, 16–18].

While some brain abnormalities, like cortical malformation, seem to be strong predictors of poor neurological outcome, the prognostic role of others, such as “white matter change,” remains unclear [19–22]. Reported studies are often small and limited to severely affected children [11, 14, 15, 17, 19, 23–26].

Furthermore, grading of the white matter can be challenging in newborns. Mainly discrete or isolated lesions can be difficult to distinguish from normal variation in the unmyelinated white matter. At present, there is no written consensus on how to define “white matter” signal alterations as pathological, leaving the (subjective) decision up to the expertise of the radiologist [21, 22].

The aim of this study was to investigate whether quantitative measurement of the white matter on brain MRI, by means of apparent diffusion coefficient (ADC) values, could be used to predict clinical outcome in newborns with cCMV infection.

## Materials and methods

Registration of this retrospective, observational study was approved by the institutional ethics committee (Health, innovation and research institute, University Hospital Ghent, Belgium) and was enlisted at the privacy commission. Children were included only after written informed consent of the parents or legal guardians.

### Study group and exclusion criteria

Data from a single tertiary center (Ghent University Hospital) was used for this study. Between April 2007 and March 2020, 286 newborns with cCMV received a brain MRI. Neonatal infection was confirmed by viral isolation and/or polymerase chain reaction (PCR) on urine or saliva within the first three weeks of life. Retrospective diagnosis (after age of 21 days) was made by PCR on dried blood spots.

For all children with cCMV, the following data were collected at enrollment: reason for diagnostic work-up, clinical features at birth, blood tests, hearing, ophthalmologic evaluation, and MRI of the brain. Patients were excluded from the study when there were comorbidities that could interfere with imaging and/or clinical findings (preterm birth, syndromes with known brain abnormalities); when brain MRI was not performed in the first 45 days of life, image quality was insufficient and/or when diffusion-weighted images (DWI) were not acquired.

### Image acquisition

MRI of the brain was performed with a head coil or neonatal head coil on a 1.5-tesla scanner (Avanto/Symphony/Aera, all manufactured by Siemens Healthineers, Erlangen, Germany). Sequences included 3–4 mm axial DWI (b0–b1000, three or four directions) with corresponding ADC mapping, 3 mm axial T2-weighted fat saturated turbo spin echo, 3 mm coronal T2-weighted turbo spin echo, 3 mm axial T1-weighted inversion recovery turbo spin echo, 3 mm sagittal T1-weighted spin echo, 3–4 mm axial T2-weighted fluid attenuated inversion recovery (FLAIR) turbo spin echo, and 3 mm axial T2*-weighted gradient echo (T2*). Detailed information on the sequences is provided in Supplementary Material 1.

### Apparent diffusion coefficient values

**Fig. 1:**
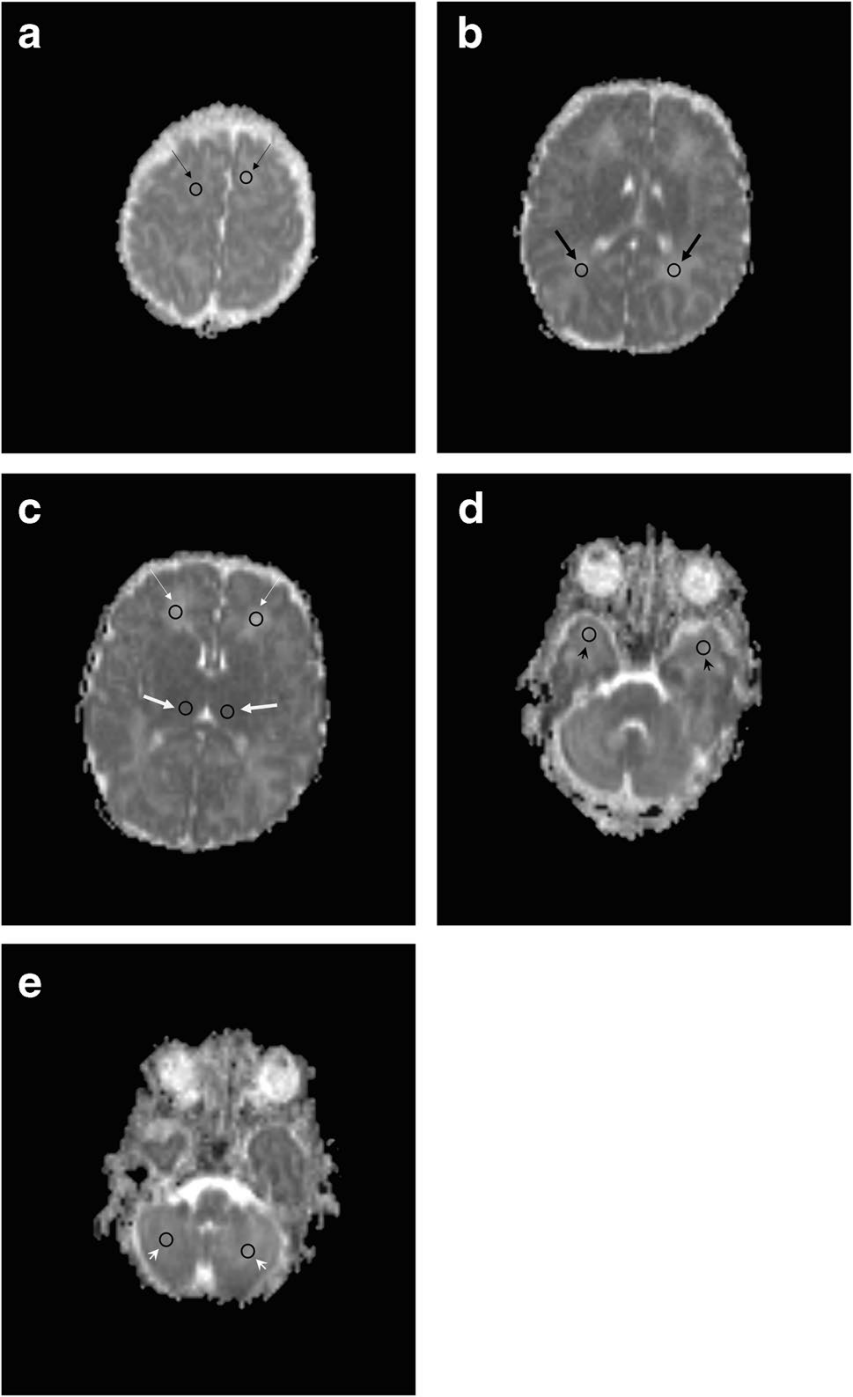
Axial apparent diffusion coefficient magnetic resonance images in a 2-week-old boy with congenital cytomegalovirus infection show placement of regions of interest (*arrows*) in the subcortical frontoparietal white matter (**a**), posterior periventricular white matter (**b**), deep anterior frontal white matter (*thin arrows* in **c**), thalami (*thick arrows* in **c**), anterior temporal white matter (**d**), and cerebellar hemispheres (**e**)

In each patient, regions of interest (ROIs) were placed systematically and symmetrically in the following white matter regions of the brain (Fig. 1): subcortical frontoparietal, posterior periventricular, deep anterior frontal, and anterior temporal white matter, both left and right. ROIs were also placed in both thalami and cerebellar hemispheres. ROIs were only placed in true brain parenchyma: when cystic lesions were present, the ROI was placed in the adjacent white matter, not including the cyst. All ROIs were positioned by a pediatric radiologist (C.V.) with 12 years of experience.

“General white matter ADC” value was calculated as the mean value of the different ROIs, placed in the above-mentioned white matter regions. ADC values were expressed in units of 10^−6^ mm^2^/s.

### Qualitative brain assessment

Two pediatric radiologists (C.V. and N.H. [with 19 years of experience]) independently reviewed the images, to assess additional qualitative brain abnormalities. Images were scored for the presence of gyral abnormalities, ventriculomegaly, and subependymal cysts. The radiologists were blinded to clinical and other imaging findings. Interobserver agreement was calculated. For further analysis, in case of disagreement, a consensus score was applied.

### Clinical follow-up

The follow-up consisted of hearing evaluation at the Otorhinolaryngology Department, vision assessment by fundoscopy, and cognitive and motor evaluation at the Center for Developmental Disorders. Periodicity of these follow-up visits conformed to the guidelines of the Flemish consensus 2018 [8]. Details are provided in Supplementary Material 2.

Neonatal hearing evaluation was carried out using automated auditory brainstem response (ABR). After referral, click-evoked ABR was performed within the first month after birth, combined with otomicroscopy and tympanometry to rule out otitis media. Neonatal ABR thresholds ≤40 dB nHL were considered normal.

Cognitive development was assessed using the Bayley Scales of Infant and Toddler Development Third Edition [27]: normal (Bayley score 86 or more), mild (70–85), moderate (50–69), and severe impairment (below 50). 

Motor development was assessed using the Alberta Infant Motor Scale (AIMS) [28]. Based on the AIMS percentile ranks, motor development was categorized into the following percentile (P) groups: above P75, between P25 and P75, between P10 and P25, and below P10. AIMS below P10 was considered abnormal [29].

### Statistical analysis

Categorical variables were expressed as number and percentage and numerical variables as median and interquartile range (IQR) or mean ± standard deviation (SD) when appropriate. Last observation carried forward analysis was used to assess the final outcome measures.

Fleiss kappa analysis was used to assess interobserver variability.

Independent *t*-tests were used to compare distributions of general white matter ADC between different outcome groups. When needed, Bonferroni-Holm correction for multiple testing was applied. A significance level of 0.05 was accepted.

After hypothesis testing, simple and elastic net regression analyses were performed. In a first step, for every outcome, a simple logistic regression model with “general white matter ADC” as an independent variable was fitted. In a second step, two model structures were crossed with the five different white matter regions (resulting in ten models per outcome).

The two model structures are as follows:

1. “White matter ADC” together with additional qualitative imaging variables (ventriculomegaly, gyral abnormalities, subependymal cysts), indicated with “extra”
2. The same as model structure 1, but with additional interaction terms of the qualitative imaging variables with “white matter ADC,” indicated with “interact”

Because of the relatively high parameter to sample size ratio, a penalized logistic regression approach was chosen, more specifically elastic net logistic regression. Elastic nets have two hyperparameters (the amount of penalization and the least absolute shrinkage and selection operator (LASSO)–ridge mixture) that can be tuned. Validation of the models was done by calculating the cross-validated receiver operating characteristic (ROC) area under the curve (AUC) and precision-recall (PR) AUC using a three times repeated fivefold cross-validation scheme. Mean performances are reported with their standard errors (SE). For the elastic nets, hyperparameter tuning was performed using 15 bootstrap samples, nested within the outer cross validation loop. The hyperparameter search was guided by a Bayesian optimization algorithm, with the validation ROC AUC as objective function. More details on the elastic net regression technique can be found in Supplementary Material 3.

For each outcome, the best performing model on the training set was selected as a final model. This final model was then refit using all available data. Variables were standardized prior to modeling in order not to let the variable scale determine the penalization.

For descriptive purposes, optimal thresholds of the simple regression analyses were calculated, based on the maximal Youden index. Using this threshold, the training positive predictive value (PPV) and negative predictive value (NPV) were calculated. These values were not cross-validated (in contrast to the ROC and PR AUC).

All analyses were performed with R version 4.2.2 (R Core Team, 2022) and the Tidymodels (Kuhn and Wickham, 2020) and glmnet (Friedman, Hastie, and Tibshirani, 2010) packages [30].

## Results

### Demographics

A total of 286 newborns with cCMV received a brain MRI. Ten patients were excluded from further evaluation: six due to comorbidities (preterm birth (3), Möbius syndrome (1), Down syndrome (1), tuberous sclerosis (1)), one because of seizure development during MRI, and three because MRI was performed after 45 days. In 21 patients, ADC was not performed, or image quality was considered insufficient for accurate ROI placement.

Of the remaining 255 included patients, 121 were male (47.5%). Median gestational age at birth was 39 weeks (IQR 38–39 weeks, missing in 11). Median age at the time of MRI was 21 days (IQR 14–28 days).

### Neonatal brain—magnetic resonance imaging

Gyral abnormalities were noted in nine (3.5%), ventriculomegaly in 24 (9.4%), and subependymal cysts in 58 (22.7%) patients.

Interobserver agreement between readers was perfect for ventriculomegaly (Kappa 1, standard error [SE] 0, 95% confidence interval [CI] 1–1), excellent for gyral abnormalities (Kappa 0.94, SE 0.06, 95% CI:0.82–1), and good for subependymal cysts (Kappa 0.76, SE 0.05, 95% CI 0.66–0.86).

### Clinical findings

Relevant clinical findings at birth and during follow-up are provided in Table 1. Reason for neonatal cCMV testing was maternal seroconversion during pregnancy in 230 patients (92.0%). Neonatal hearing was abnormal in 14 patients (5.6%). Impaired cognitive development was noted in 18 patients (7.4%) and impaired motor development in 35 (14.6%). Median clinical follow-up was 12 months (IQR 12–27 months).

**Table 1:** Relevant clinical findings at the time of neonatal magnetic resonance imaging and during clinical follow-up

	Frequency (*n*)	Valid %
**Reason for cCMV testing**		
Maternal seroconversion	230	92.0
Standard screening	5	2.0
Small for gestational age	3	1.2
Hematologic abnormalities	2	0.8
Abnormal prenatal imaging	1	0.4
Other (cholestasis, microcephaly, seizures,…)	9	3.6
Missing/unknown	5	
**Hearing evaluation at birth (neonatal hearing)**		
Normal	236	94.4
Unilateral hearing loss	10	4.0
Bilateral hearing loss	4	1.6
Missing	5	
**Cognitive development—Bayley score**		
Normal (≥ 86)	224	92.6
Mild impairment (70–85)	14	5.8
Moderate impairment (50–69)	2	0.8
Severe impairment (< 50)	1	0.4
Deceased	1	0.4
Missing/unknown	13	
**Motor development—AIMS (percentile [P])**		
> P75	62	25.9
P25–7P5%	101	42.3
P10–P25%	41	17.2
< P10%	35	14.6
Missing/unknown	16	
**Age at last clinical follow-up**		
4–5 months	20	8.5
6–11 months	37	15.7
12–17 months	99	41.9
18–23 months	6	2.5
2–3 years	43	18.2
4 years or more	31	13.1
Missing/unknown	19	
**Total patients (*n*)**	255	

*AIMS* Alberta Infant Motor Scale, *cCMV* congenital cytomegalovirus

### Hypothesis testing

General white matter ADC was significantly higher (*P*<0.05) in patients with neonatal hearing loss and impaired cognitive and motor development: mean ADC value was 1,801.9 (± SD 177.7) in patients with hearing loss, compared to 1,626.4 (± 189.2) with normal hearing (mean difference 175.5, *P*=0.011). Mean ADC value was 1,748.3 (± 187.2) in patients with cognitive impairment compared to 1,628.0 (± 192.7) with normal cognition (mean difference 120.2, *P*=0.039). Mean value was 1,742.1 (± 276.8) in patients with motor impairment versus 1,616.4 (± 170.9) with normal motor development (mean difference 125.7, *P*=0.039).

No significant differences were seen in the ADC values of the cerebellum and the thalami, according to clinical outcome. Details are provided in Table 2.

**Table 2:** Hypothesis tests—apparent diffusion coefficient values in the white matter, thalami, and cerebellum for different outcome groups

		Normal		Abnormal		*P*-value^a^
		Mean value	SD	Mean value	SD	
General white matter ADC	Hearing	1626.4	189.2	1801.9	177.7	***0.011***
	Cognitive	1628.0	192.7	1748.3	187.2	***0.039***
	Motor	1616.4	170.9	1742.1	276.8	***0.039***
Thalamus ADC	Hearing	1034.4	59.7	1023.7	61.9	0.539
	Cognitive	1036.6	59.2	1015.7	81.9	0.302
	Motor	1038.4	60.3	1018.5	63.4	0.092
Cerebellum ADC	Hearing	1140.3	110.3	1221.4	161.7	0.086
	Cognitive	1142.3	107.1	1177.8	183.7	0.429
	Motor	1144.3	109.4	1134.7	126.3	0.674

^a^Bold represents statistical significance (*P*<0.05)

*ADC* apparent diffusion coefficient, *SD* standard deviation

### Modeling

#### Neonatal hearing

The simple logistic regression model using general white matter had a ROC AUC of 0.79 (SE 0.03). Optimal probability threshold for this model based on the Youden index was 0.0497 (specificity 0.62, sensitivity 0.86), with a general white matter ADC value of 1673 or more predicting hearing loss (PPV 0.11, NPV 0.99).

**Fig. 2:**
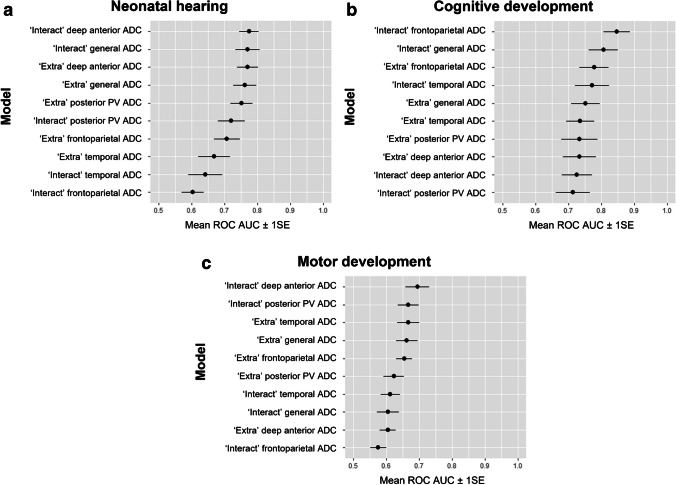
Results of the ten elastic net models per outcome: neonatal hearing (**a**), and cognitive (**b**) and motor (**c**) development. For each model, mean receiver operating characteristic area under the curve (mean ROC AUC) ± 1 standard error (SE) is shown. *ADC *apparent diffusion coefficient, *PV* posterior periventricular

**Fig. 3:**
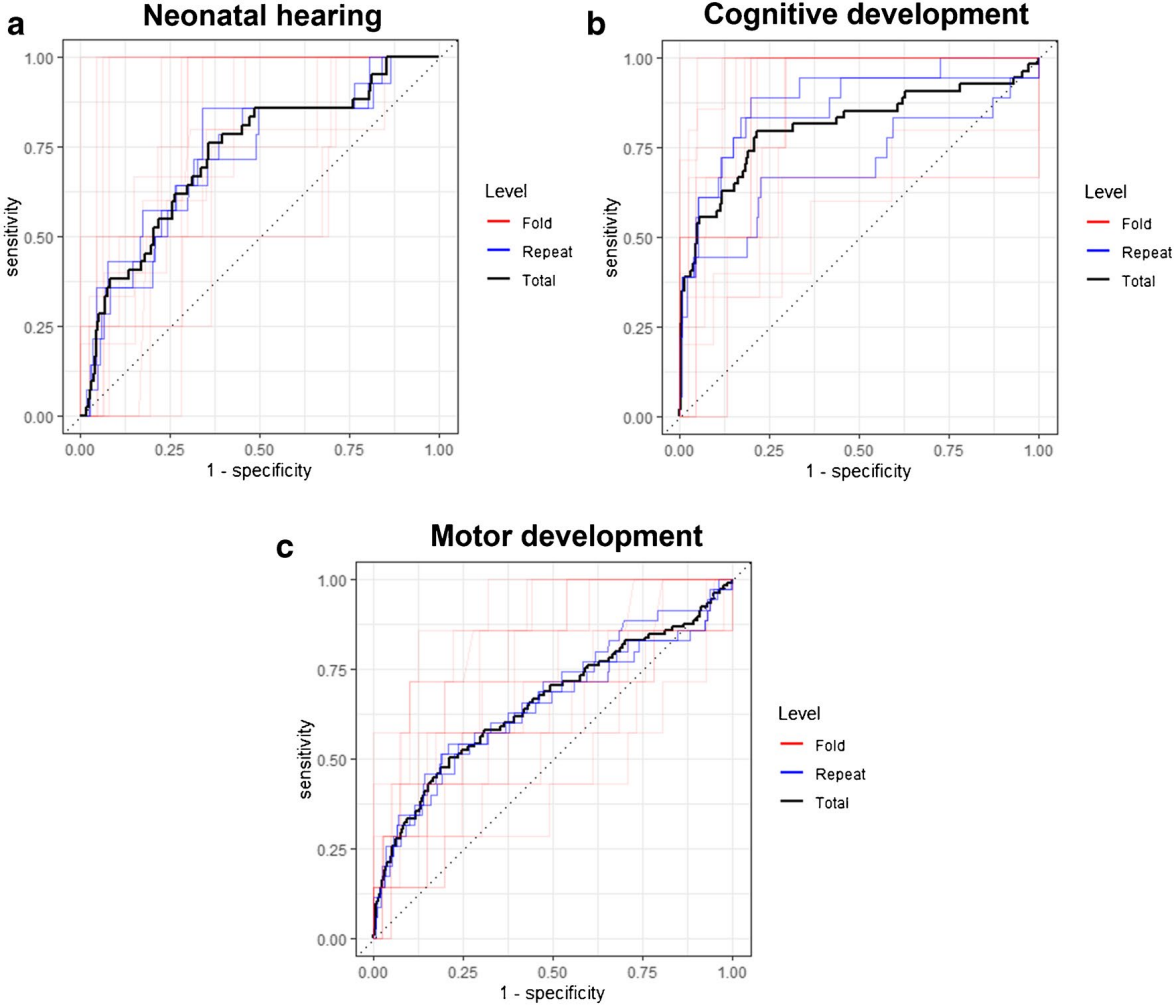
Cross-validated receiver operating characteristic curves for neonatal hearing (**a**), and cognitive (**b**) and motor (**c**) development

The results of the ten elastic net models are summarized in Fig. 2 (details in Supplementary Material 4). The simple logistic model was not improved by including qualitative imaging findings or by using ADC in the separate brain regions in the elastic net analysis: the best performing elastic net model was the interaction model in the deep anterior frontal region, with AUC 0.77 (SE 0.03). The cross-validated ROC curves are displayed in Fig. 3. Since the simple logistic regression performed best, it was chosen as the final model. This final model had an AUC on the training set of 0.76. The coefficients of the final model and necessary statistics to calculate and the ROC table are respectively provided in Supplementary Materials 5 and 6.

#### Cognitive development

Simple logistic regression using general white matter had ROC AUC 0.74 (SE 0.02). Optimal probability threshold was 0.0671 (specificity 0.58, sensitivity 0.83), with a general white matter ADC value of at least 1,644 predicting cognitive impairment (PPV 0.14, NPV 0.98).

Elastic net analysis (with inclusion of qualitative imaging findings) provided a higher AUC (Figs. 2 and 3, Supplementary Material 4): the two best performing models were both frontoparietal—the interaction model in the frontoparietal region having the highest AUC of 0.85 (SE 0.04)—therefore, it was chosen as the final model (Supplementary Material 5). This final model had an AUC on the training set of 0.89 (Supplementary Material 6).

#### Motor development

Simple logistic regression using general white matter had ROC AUC 0.64 (SE 0.04). Optimal probability threshold was 0.159 (specificity 0.72, sensitivity 0.57), with a general white matter ADC value of a least 1,707 predicting motor impairment (PPV 0.26, NPV 0.91).

Elastic net analysis (with inclusion of qualitative imaging findings) provided a higher AUC (Figs. 2 and 3, Supplementary Material 4): the best performing elastic net model was the interaction model in the deep anterior frontal region, with the highest AUC of 0.69 (SE 0.04)—therefore, it was chosen as a final model (Supplementary Material 5). This final model had an AUC on the training set of 0.73 (Supplementary Material 6).

## Discussion

In this study, we investigated the association between quantitative measurement of white matter ADC values and clinical outcome in newborns with cCMV. ADC was significantly higher in patients with neonatal hearing loss and with cognitive and motor impairment during follow-up. Quantitative ADC measurement provided a valuable prediction tool, with ROC AUC of the best performing logistic regression models ranging between 0.73 and 0.89.

To our knowledge, this is the first study examining white matter ADC in a cohort of cCMV-infected newborns. A few smaller studies investigated DWI in fetal MRI. Kotovich et al. found reduced ADC values in most brain areas of 90 CMV-infected fetuses, compared to a healthy control group. No correlation was found between ADC and neurocognitive development [31]. Aertsen et al., however, recently found significantly higher ADC values in the white matter of 46 infected fetuses compared to a seronegative control group, while ADC in the basal ganglia and cerebellum did not differ significantly. They also found higher ADC values in the temporal and frontal white matter of infected fetuses with severe brain abnormalities, compared to fetuses with mild abnormalities [32]. Although these studies were based on fetal (rather than neonatal) MRI, our findings are in support of the latter study. We also found higher ADC values in the white matter of patients with clinical impairments, while ADC in the thalami and cerebellum did not differ significantly.

For predicting neonatal hearing loss, simple logistic regression using only “general white matter ADC” was the best model in our study, with AUC almost reaching 0.8. This association between white matter and clinical features in the neonatal setting strengthens the belief that white matter signal alterations are indeed part of the symptomatic spectrum of cCMV [17], and not merely variations in normal myelination.

The mechanism of hearing loss in cCMV is not well understood. Hearing loss can be present at birth, but it can also develop later in life and can occur in both initially symptomatic and asymptomatic children [9]. A recent study showed no clear association between brain abnormalities on MRI and late-onset hearing loss [33]. One hypothesis is that late-onset hearing loss may be caused by an immune-mediated delayed endolymphatic hydrops. As late-onset hearing loss may be caused by a different mechanism and the precise mechanism is unknown, we decided not to include this possible confounding information. Therefore, we only looked at early hearing loss, although this might be a subject for future research [34].

Cognitive developmental problems occur in a minority (about 10%) of patients with cCMV; however, they are a serious complication [6, 7, 29]. While some previous studies found white matter abnormalities valuable for predicting neurodevelopmental outcome [12, 35, 36], others found them unreliable [19, 23]. In our study, simple logistic regression using only general white matter ADC could predict cognitive impairments with AUC reaching 0.74; the prediction model was improved (with AUC almost reaching 0.9) by including interaction with other brain abnormalities. Also, a better model was found when using frontoparietal ADC, compared to general white matter or other regions of the brain. This could possibly be explained by the affected regions playing a more important role in cognitive functioning.

Although general white matter ADC was significantly higher in patients with impaired motor development, simple logistic regression “only” had an AUC of 0.64. Prediction could be improved by including interaction with other brain abnormalities and by using ADC in the deep anterior frontal white matter (AUC exceeding 0.7). In interpreting the slightly worse capability of predicting motor delay, we need to take into account the rather short follow-up of several patients in this study. In the first 18 months of life, a large variance in normal motor development can be observed. Problems later in life might not be picked up in the first few months. On the other hand, mild hypotonia early in life can influence the AIMS score, without persistent problems at a later age. Larger and longer follow-up studies are needed to fine-tune this prediction model.

This study shows that white matter ADC, in combination with other qualitative imaging variables, allows a fairly good distinction between children with or without clinical impairments (AUC between 0.73 and 0.89). The AUC of the models were cross-validated: as these were calculated on data not previously seen by the model, they provide an indication of the generalizability. For predicting hearing loss or neuromotor impairments, we found ADC cutoff values ranging between 1,650 and 1,700. These values are largely in line with other studies investigating changes in ADC during normal brain myelination or in patients with other white matter disease [37–39]. However, we wish to emphasize that these thresholds are not cross-validated and therefore only serve descriptive purposes. Longer follow-up and larger cohorts are necessary, as well as external validation of the suggested models to define clear cutoff values.

Our study has some limitations. Although to our knowledge, this is one of the first cohorts studying the predictive value of neonatal white matter ADC in cCMV, the number of patients with detected problems might still be too small for robust conclusions. Also, the length of follow-up in several patients was probably too short. During follow-up, only the last observation carried forward was noted.

Therapy could have caused a bias in developmental outcome. The use of multiple scanners (with different signal to noise ratios) and (slightly) different sequences could have been a confounding factor in interpreting ADC values. Another limitation was the lack of a control group of normal newborns. However, obtaining ethical approval and finding parents to consent for an MRI without any clinical need would be difficult.

## Conclusion

In neonates with cCMV, white matter ADC, in combination with other qualitative imaging variables, allows a fairly good distinction between children with or without clinical impairments. Neonatal MRI with quantitative ADC measurement may be a useful tool for guiding therapy and providing accurate parent counseling in cCMV infection.

### Supplementary Information

Below is the link to the supplementary material.

Supplementary file1 (DOCX 67.0 KB)

## Data Availability

Raw data are not publicly available to preserve individuals’ privacy under the European General Data Protection Regulation. All data relevant to the study are included in the article or uploaded as online supplemental information.
