# Supplementary material for: Apparent diffusion coefficient values of the white matter in magnetic resonance imaging of the neonatal brain may help predict outcome in congenital cytomegalovirus infection
Source: Pediatr Radiol. 2024 Jan 6;54(2):337–46. doi: 10.1007/s00247-023-05838-9 (PMC10830666; doi:10.1007/s00247-023-05838-9)
Supplement: Supplementary file 1 — Supplementary file1 (DOCX 67.0 KB) [file 247_2023_5838_MOESM1_ESM.docx]

**Supplementary material 1:**

***Imaging sequences used for magnetic resonance imaging of the brain in newborns with congenital cytomegalovirus infection***

| **Scan sequence** |  | **ST (mm)** | **Matrix** | **TR**  **(msec)** | **TE**  **(msec)** | **TI**  **(msec)** | **Flip angle (deg)** | **Sequence**  **type** |
| --- | --- | --- | --- | --- | --- | --- | --- | --- |
| **Axial DWI** (b0-1000) |  | 3-4 | 128 | 3400-4900 | 85-94 | NA | 90 | EP |
|  | *or* | 3 | 128 | 6930-7350 | 82-83 | NA | 180 | EP |
| **Sagittal T1** |  | 3 | 256 | 500-550 | 7,8-14 | NA | 90 | SE |
| **Axial T2** |  | 3 | 384 | 5400-9890 | 72-118 | NA | 150-180 | SE FS |
| **Axial T1** |  | 3-4 | 256 | 485-500 | 10-14 | NA | 70 | SE |
|  | *or* | 3-4 | 256 | 4770-7000 | 55-60 | 350-400 | 150-160 | SE IR |
| **Axial flair** |  | 3-4 | 256 | 9000 | 108-114 | 2500 | 150 | SE IR |
|  | *or* | 3 | 256 | 9000 | 84 | 2500 | 150 | SE IR FS |
| **Axial T2*** |  | 3-4 | 256 | 800-1160 | 25-26 | NA | 20 | GR |
| **Coronal T2** |  | 3 | 512 | 3000-7050 | 76-98 | NA | 150-180 | SE |

*DWI* diffusion weighted imaging*, EP* echo planar imaging, *flair* fluid attenuated inversion recovery*, FS* fat saturated, GR gradient echo, *IR* inversion recovery, *NA* not applicable, *ST* slice thickness*, TE* echo time*, TI* inversion time*,* TR repetition time*, SE* spin echo

**Supplementary material 2*:***

***Recommended follow-up for children with congenital cytomegalovirus infection in Flanders* [1]**

|  | Birth | 3-4 months | 6 months | 1 year | 1.5 year | 2 years | 2.5 years | 3 years | 4  years | 4.5 years | 5  years | 6 years |
| --- | --- | --- | --- | --- | --- | --- | --- | --- | --- | --- | --- | --- |
| Vision | X1 |  |  | X1 |  | X1 |  | X1 | X1 |  | X1 | X1 |
| Hearing | X2 | X2 | X2 | X2 | X2(S) | X2 | X2(S) | X2 | X2 |  | X2 | X2 |
| Cognitive/motor  development |  | X3(S) |  | X3 | X3(S) | X3(IR) |  | X3(IR) |  | X3(IR) |  | Q |

*X1: follow-up by ophthalmologist (fundoscopy)*

*X2: follow-up by ENT specialist*

*X3: follow-up by Center for Developmental Disorders*

*(S): only in symptomatic children*

*(IR): if required by specialists*

*Q: by questionnaire sent to parents*

**Reference list**

1. Keymeulen A, De Leenheer E, Goderis J, Dhooge I, Smets K, Flemish Society of Pediatrics N, Perinatal Epidemiology Working Group for c CMVi (2019) Congenital cytomegalovirus infection registry in flanders: opportunities and pitfalls. Acta clinica Belgica:1–8

**Supplementary Material 3*:***

***A Gentle Introduction to Elastic Net Regression***

For the sake of simplicity, the following introduction covers elastic net regression in the context of linear regression. However, the concepts extend naturally to generalized linear regression, for example logistic regression.

When building a (predictive) regression model, one is faced with several challenges of which the prevention of overfitting is a very important one [1]. A model is a product of the sample data on which it was built. We can think of 3 situations. The first one is where the model is not complex enough and is unable to extract enough information from the data. Because of this lack of extacted information, it generalizes poorly to new observations. In the second situation, the model is overly complex and fits the data extremely well so that it has incorporated not only the information but also the idiosyncratic noise that is present in the data. The result is also a poorly performing model, but now because of a different reason. This model generalizes poorly to new data because the idiosyncrasies it learnt so well are not useful for new observations. The third and ideal situation is where the model is complex enough so that the relevant signals are extracted, yet still simple enough so that it only incorporates signals and no noise. As a result, a model like this will generalize well to new observations.

In practice, one way to achieve model complexity is by entering multiple potentially important variables into the model. This can be problematic when we are uncertain of the association between the predictors and the response variable. Also, for a fixed sample size, the risk of overfitting becomes larger as the predictor set grows, especially when unrelated, noisy, or sometimes very strongly interrelated variables are entered into the model. This is all related to the problem of model selection: which (combination of) variables should we include?

One way to deal with potential overfitting is to use penalized regression (sometimes also called regularized regression) [2]. LASSO regression is a penalized regression technique that achieves a limited model complexity by shrinking some coefficients down towards zero and by letting some coefficients be exactly equal to 0. The result of the latter is that the LASSO performs model selection, which can be desirably in scenarios where predictors of which the association with the response is unclear. Ridge regression is another penalized regression technique that achieves a limited model complexity by shrinking the coefficients down towards but never exactly zero. This can be desirable when we want to keep all variables in the model and when the variables are strongly interrelated.

Choosing the ridge or the LASSO penalty requires knowing which is best for the given situation, which is often difficult. Also, both approaches have appealing properties and we may want to have both the selection properties of the LASSO and the shrinkage properties of the ridge. This is where elastic net comes in as a combination of both penalization methods [3]. In the case of the elastic net, it is possible to choose the degree of LASSO penalty, relative to the ridge penalty. However, it is customary to make this choice (including the overall degree of penalization) through cross validation. As such, the elastic net is a versatile method as it is able to adapt to the particular characteristics of the problem and data at hand.

In conclusion, elastic net is a type of penalized regression where a mixture of the LASSO and ridge penalty is used. It is a powerful tool to build robust prediction models, especially in situations where we have many potentially important (and interrelated) variables and where the sample size is low relative to the number of variables.

**Reference list**

1. Hastie, T., Tibshirani, R. and Friedman, J. (2009) The Elements of Statistical Learning: Data Mining, Inference, and Prediction. 2nd Edition, Springer, New York. <https://doi.org/10.1007/978-0-387-84858-7>
2. Friedrich S, Groll A, Ickstadt K, Kneib T, Pauly M, Rahnenfuhrer J, Friede T (2023) Regularization approaches in clinical biostatistics: A review of methods and their applications. Statistical methods in medical research. <https://doi.org/10.1177/09622802221133557>
3. Zou H, Hastie T (2005) Regularization and Variable Selection Via the Elastic Net. J R Stat Soc Series B Stat Methodol, 67:301–320. <https://doi.org/10.1111/j.1467-9868.2005.00503.x>

**Supplementary material 4:**

***Detailed results of the ten elastic net models for different outcome measures***

|  | **NEONATAL**  **HEARING** | | | | **COGNITIVE**  **DEVELOPMENT** | | | | **MOTOR**  **DEVELOPMENT** | | | |
| --- | --- | --- | --- | --- | --- | --- | --- | --- | --- | --- | --- | --- |
|  | **PR AUC** | | **ROC AUC** | | **PR AUC** | | **ROC AUC** | | **PR AUC** | | **ROC AUC** | |
| **SET** | **Est** | **SE** | **Est** | **SE** | **Est** | **SE** | **Est** | **SE** | **Est** | **SE** | **Est** | **SE** |
| **General white matter** | | | | | | | | | | | | |
| **extra** | 0.292 | 0.061 | 0.761 | 0.035 | 0.476 | 0.071 | 0.751 | 0.044 | 0.384 | 0.037 | 0.662 | 0.032 |
| **interact** | 0.295 | 0.056 | 0.770 | 0.036 | 0.518 | 0.080 | 0.806 | 0.045 | 0.360 | 0.037 | 0.605 | 0.033 |
| **Deep anterior white matter** | | | | | | | | | | | | |
| **extra** | 0.241 | 0.062 | 0.770 | 0.032 | 0.520 | 0.069 | 0.733 | 0.050 | 0.372 | 0.037 | 0.604 | 0.024 |
| **interact** | *0.234* | *0.048* | ***0.774*** | *0.029* | 0.422 | 0.065 | 0.726 | 0.046 | 0.391 | 0.039 | ***0.694*** | 0.036 |
| **Frontoparietal white matter** | | | | | | | | | | | | |
| **extra** | 0.312 | 0.059 | 0.707 | 0.039 | 0.407 | 0.062 | 0.778 | 0.044 | 0.367 | 0.038 | 0.654 | 0.024 |
| **interact** | 0.288 | 0.054 | 0.603 | 0.034 | 0.498 | 0.079 | ***0.847*** | 0.040 | 0.343 | 0.039 | 0.575 | 0.025 |
| **Posterior periventricular white matter** | | | | | | | | | | | | |
| **extra** | 0.256 | 0.057 | 0.752 | 0.033 | 0.511 | 0.065 | 0.733 | 0.055 | 0.393 | 0.041 | 0.623 | 0.031 |
| **interact** | 0.227 | 0.049 | 0.721 | 0.041 | 0.395 | 0.074 | 0.714 | 0.052 | 0.386 | 0.038 | 0.667 | 0.032 |
| **Temporal white matter** | | | | | | | | | | | | |
| **extra** | 0.294 | 0.058 | 0.668 | 0.048 | 0.478 | 0.071 | 0.736 | 0.042 | 0.352 | 0.041 | 0.666 | 0.033 |
| **interact** | 0.264 | 0.053 | 0.642 | 0.052 | 0.490 | 0.078 | 0.771 | 0.052 | 0.325 | 0.040 | 0.612 | 0.029 |

*AUC* Area Under the Curve*, Est* Estimate, *PR* Precision Recall*, ROC* Receiver Operating Characteristics*, SE* Standard Error

**Supplementary material 5: *Final models – coefficients and necessary statistics to calculate***

| **NEONATAL HEARING** | | | | | |
| --- | --- | --- | --- | --- | --- |
| **Coefficients** | | | | | |
|  | **term** | **estimate** | **SE** | **statistic** | **p.value** |
|  | (Intercept) | -3.092 | 0.330 | 9.355 | 8.379^e^-21 |
|  | ADC general white matter | 0.745 | 0.240 | -3.099 | 1.944^e^-03 |
| **Calculation of z-scores** | | | | | |
|  | **term** | **mean** | **SD** |  |  |
|  | ADC general white matter | 1636.21 | 192.52 |  |  |
| **Formula:** | | | | | |
| *Z = ( -3.092 ) + ( 0.745 x ADC general white matter-zscore)*  *P(Y=1\|X=x) = 1/(1 + exp(-Z))* | | | | | |
| **COGNITIVE DEVELOPMENT** | | | | | |
| **Coefficients** | | | | *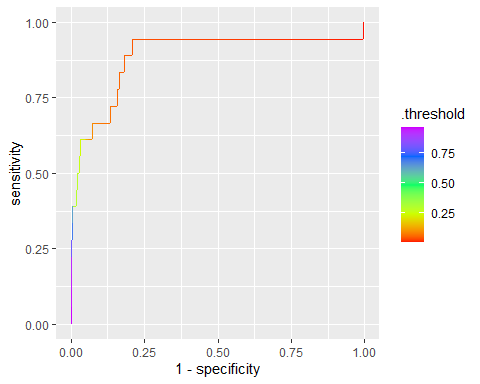****ROC curve of the final model*** | |
|  | **term** | **estimate** |  |  |  |
|  | (Intercept) | -3.027 |  |  |  |
|  | ADC mean frontoparietal WM | 0.492 |  |  |  |
|  | Ventriculomegaly | 0.736 |  |  |  |
|  | Gyral abnormalities | 0.542 |  |  |  |
|  | ADC mean frontoparietal WM_ x_subependymal cysts | -0.354 |  |  |  |
|  | ADC mean frontoparietal WM_ x_gyral abnormalities | -0.212 |  |  |  |
| **Calculation of z-scores** | | | |  |  |
|  | **term** | **mean** | **SD** |  |  |
|  | ADC mean frontoparietal WM | 1570.16 | 227.14 |  |  |
|  | Cysts | 0.23 | 0.42 |  |  |
|  | Ventriculomegaly | 0.10 | 0.29 |  |  |
|  | Gyral abnormalities | 0.04 | 0.19 |  |  |
| **Formula:** | | | | | |
| *Z = (-3.027) + (0.492 x ADC mean frontoparietal WM-zscore) + (0.736 x ventriculomegaly-zscore) + (0.542 x gyral abnormalities-zscore) + (-0.354 x ADC mean frontoparietal WM_x_subependymal cysts-zscore) + (-0.212 x ADC mean frontoparietal WM_x_gyral abnormalities-zscore)*  *P(Y=1\|X=x) = 1/(1 + exp(-Z))* | | | | | |
| **MOTOR DEVELOPMENT** | | | | | |
| **Coefficients** | | | | ***ROC curve of the final model***  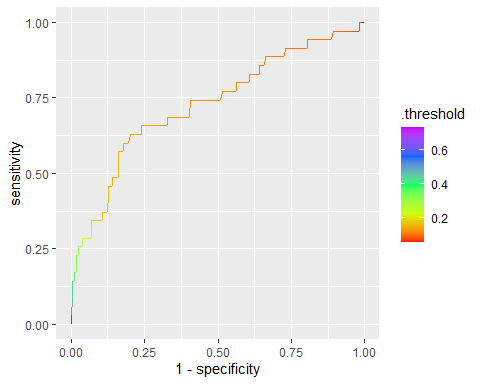 | |
|  | **term** | **estimate** |  |  |  |
|  | (Intercept) | -1.974 |  |  |  |
|  | Subependymal cysts | 0.006 |  |  |  |
|  | Ventriculomegaly | 0.114 |  |  |  |
|  | Gyral abnormalities | 0.094 |  |  |  |
|  | ADC mean deep anterior WM_  x _subependymal cysts | 0.343 |  |  |  |
| **Calculation of z-scores** | | | |  |  |
|  | **term** | **Mean** | **SD** |  |  |
|  | ADC mean deep anterior WM | 1706.27 | 200.49 |  |  |
|  | Subependymal cysts | 0.23 | 0.42 |  |  |
|  | Ventriculomegaly | 0.09 | 0.29 |  |  |
|  | Gyral abnormalities | 0.03 | 0.18 |  |  |
| **Formula:** | | | | | |
| *Z = (-1.974) + (0.006 x subependymal cysts-zscore) + (0.114 x ventriculomegaly-zscore) + (0.094 x gyral abnormalities-zscore) + (0.343 x ADC mean deep anterior WM_x_subependymal cysts-zscore)*  *P(Y=1\|X=x) = 1/(1 + exp(-Z))* | | | | | |

*SD* standard deviation, *SE* Standard error*, WM* white matter

**Supplementary material 6:**

***Final models – Receiver Operating Characteristics tables***

| **NEONATAL HEARING** | | | **COGNITIVE DEVELOPMENT** | | | **MOTOR DEVELOPMENT** | | |
| --- | --- | --- | --- | --- | --- | --- | --- | --- |
| ***threshold*** | ***specificity*** | ***sensitivity*** | ***threshold*** | ***specificity*** | ***sensitivity*** | ***threshold*** | ***specificity*** | ***sensitivity*** |
| 0.027 | 0.280 | 1.000 | 0.010 | 0.004 | 1.000 | 0.084 | 0.020 | 1.000 |
| 0.029 | 0.331 | 0.929 | 0.046 | 0.790 | 0.944 | 0.099 | 0.108 | 0.971 |
| 0.050 | 0.623 | 0.857 | 0.054 | 0.817 | 0.889 | 0.105 | 0.196 | 0.943 |
| 0.051 | 0.636 | 0.786 | 0.058 | 0.835 | 0.833 | 0.109 | 0.270 | 0.914 |
| 0.056 | 0.682 | 0.714 | 0.060 | 0.844 | 0.778 | 0.113 | 0.338 | 0.886 |
| 0.070 | 0.775 | 0.643 | 0.070 | 0.866 | 0.722 | 0.116 | 0.392 | 0.829 |
| 0.079 | 0.843 | 0.500 | 0.097 | 0.929 | 0.667 | 0.119 | 0.436 | 0.800 |
| 0.094 | 0.886 | 0.429 | 0.243 | 0.969 | 0.611 | 0.121 | 0.485 | 0.771 |
| 0.117 | 0.936 | 0.357 | 0.261 | 0.973 | 0.556 | 0.130 | 0.593 | 0.743 |
| 0.121 | 0.941 | 0.286 | 0.288 | 0.978 | 0.500 | 0.134 | 0.672 | 0.686 |
| 0.140 | 0.970 | 0.214 | 0.296 | 0.982 | 0.444 | 0.140 | 0.760 | 0.657 |
| 0.178 | 0.975 | 0.143 | 0.628 | 0.996 | 0.389 | 0.145 | 0.799 | 0.629 |
| 0.218 | 0.983 | 0.071 | 0.797 | 1.000 | 0.278 | 0.151 | 0.824 | 0.600 |
| - | 1.000 | 0.000 |  |  |  | 0.153 | 0.838 | 0.571 |
|  |  |  |  |  |  | 0.157 | 0.858 | 0.486 |
|  |  |  |  |  |  | 0.159 | 0.873 | 0.457 |
|  |  |  |  |  |  | 0.163 | 0.877 | 0.400 |
|  |  |  |  |  |  | 0.170 | 0.892 | 0.371 |
|  |  |  |  |  |  | 0.209 | 0.931 | 0.343 |
|  |  |  |  |  |  | 0.252 | 0.961 | 0.286 |
|  |  |  |  |  |  | 0.305 | 0.975 | 0.257 |
|  |  |  |  |  |  | 0.313 | 0.980 | 0.229 |
|  |  |  |  |  |  | 0.367 | 0.990 | 0.171 |
|  |  |  |  |  |  | 0.412 | 0.995 | 0.143 |
|  |  |  |  |  |  | 0.507 | 1.000 | 0.057 |
